# Parenteral nutrition in advanced cancer: A qualitative study on decision-making and information needs of patients and carers

**DOI:** 10.1371/journal.pone.0350396

**Published:** 2026-06-02

**Authors:** Jennifer McCracken, Sally Wheelwright, Clare Shaw

**Affiliations:** 1 Department of Nutrition and Dietetics, The Royal Marsden NHS Foundation Trust, London and Sutton, United Kingdom; 2 Department of Clinical and Experimental Medicine, Brighton and Sussex Medical School, University of Brighton and University of Sussex, Brighton, United Kingdom; 3 Biomedical Research Centre at The Royal Marsden and Institute of Cancer Research, London, United Kingdom; Queensland University of Technology - QUT: Queensland University of Technology, AUSTRALIA

## Abstract

**Objectives:**

To identify the information needs of people with advanced cancer, and their carers, to make an informed decision to commence or discontinue parenteral nutrition (PN).

**Methods:**

Semi-structured interviews with people who had advanced cancer and were receiving PN, and their informal carers were audio-recorded with consent and transcribed verbatim. Analysis was conducted using a framework analysis approach. Patients were recruited via four hospitals including a cancer centre and intestinal failure units. Carers were recruited via recruiting hospitals, advertisement on social media and support group forums.

**Results:**

Interviews were conducted with five patients and six carers. Five overarching themes were identified: factors affecting the decision: lack of choice and the importance of hope and advocacy, communication and information: whose role is it?, tackling discussions around benefits, risks and challenges of PN, the reality of living with home PN and neglected conversations: stopping PN and advance care planning. Patients and carers suggested essential information provided should include how to recognise complications, what to expect with home PN, and the risks and benefits of PN. They also recommended ways to improve service delivery including identification of the professional responsible for PN, improving communication through multiprofessional meetings and establishing a clear home PN pathway and service specification.

**Conclusions:**

This study has identified information that patients with advanced cancer and their carers need to make decisions around commencing and discontinuing parenteral nutrition. This knowledge can contribute to the development of decision tools to support shared decision-making among patients, carers and healthcare professionals.

## Introduction

The use of parenteral nutrition (PN) in advanced (incurable) cancer has increased in recent years, with people with cancer making up 1 in 4 new home PN (HPN) registrations [1]. In the UK, PN is more likely to be considered for people with advanced cancer who also have intestinal failure [2,3]. This may be a consequence of cancer or its treatment, such as malignant bowel obstruction (partial, intermittent or complete), short bowel following surgery, enterocutaneous fistula, dysmotility or severe mucosal disease due to chemotherapy or radiotherapy [1]. European guidelines state that HPN should be considered for people with advanced (incurable) cancer if survival is limited more by under-nutrition than tumour progression [4]. HPN should be offered to people with advanced cancer and intestinal failure if enteral nutrition is insufficient, estimated prognosis due to tumour progression is > 2–3 months, PN is expected to stabilise or improve performance status and quality of life or if the patient wishes to receive PN [4].

Given the complexity of HPN decision-making, it is recommended that healthcare professionals (HCPs) should take a personalised approach, considering patient and family wishes, home circumstances and treatment plans [5]. Shared decision-making is a key component of personalised care [6]. The National Institute for Health and Care Excellence (NICE) recommend that shared decision-making is actively promoted and define this as, ‘a collaborative process that involves a person and their healthcare professional working together to reach a joint decision about care’ [7]. However, research has found patients and carers do not always feel involved in decision-making around PN, despite their oncologist believing they facilitated shared decision-making [8]. The rationale for stopping PN also presents some clinical dilemmas and is not always discussed with patients [8], although it is recommended reasons for withdrawal should be discussed on initiation of PN [9].

Our scoping review showed no studies have specifically explored the information needs of people with advanced cancer, or their carers, to facilitate their decision-making around PN [10]. The aim of this study was therefore to identify the information needs of people with advanced cancer, and their carers, to enable them to make shared decisions around commencing and discontinuing PN.

## Methods

### Study design

This qualitative study comprised semi-structured interviews with patients and carers. Interview topic guides (S1 Appendix) were developed by the lead researcher and reviewed by the study advisory group, which included a patient and public involvement (PPI) contributor and experts from clinical and research backgrounds, including palliative care clinicians, a clinical-academic nurse and dietitian. The patient and carer interview topic guides were each piloted by a PPI contributor and adapted accordingly.

### Participants

Participants were adults aged ≥ 18 years, from the following groups:

- Patients with advanced cancer receiving PN
- Patients with advanced cancer making a decision to commence, or discontinue PN
- Carers of patients with advanced cancer receiving or who have received PN
- Carers of patients with advanced cancer making a decision to commence, or discontinue PN

Carers did not have to be those caring for a patient participant.

### Recruitment

Patients were recruited from one of four recruiting hospitals in Greater London from 9.6.21 to 30.6.22. These included a cancer centre and intestinal failure centres. Purposive sampling was used. Carers were recruited via the recruiting hospitals and an advertisement shared via the British Dietetic Association’s oncology specialist group Twitter (now X) and cancer and PN support group forums/ newsletters (Bowel Cancer UK, Target Ovarian Cancer, PINNT). The research team collaborated with a PPI contributor to film a recruitment video aimed at carers, which was shared via Twitter/ X (@BDA_Oncology).

### Data collection

Demographic and clinical data was gathered from all patient participants medical records with their written consent (Table 1). Semi-structured interviews were conducted by two authors (JM & CS). Flexible use of interview topic guides allowed new topics to be explored during the interview as guided by the participant. Participants were asked questions about what they knew about PN, decision making and what information they had been given (S1 Appendix). Participants were interviewed on one occasion but were invited to contact the research team if they wanted to add anything post-interview. Ten interviews were conducted remotely via telephone or Microsoft Teams due to the COVID-19 pandemic, and one was conducted during an inpatient admission. All interviews were audio-recorded with participants written consent and transcribed verbatim. Interviews lasted up to 1.5hours. Field notes were made during the interview process to aid subsequent analysis and reflexivity.

**Table 1:** Patient characteristics.

	Patient (n = 5)
Age, median (range)	50 (40-82)
Diagnosis	
Mucinous goblet cell adenocarcinoma	1
Colorectal cancer	1
Myeloma	1
Metastatic GOJ adenocarcinoma	1
Endometrioid adenocarcinoma	1
ECOG performance status	
0	2
1	–
2	2
3	1
4	–
Current treatment	
Palliative chemotherapy	2
Hormonal therapy	1
Monoclonal antibody + Dexamethasone	1
No treatment	1
Reason for commencing PN	
Bowel obstruction	4
Pan-intestinal dysmotility	1
Length of time on PN	
Less than 3 months	1
3–6 months	2
6–12 months	–
1–2 years	–
2–5 years	1
> 5 years	1
Is there documented discussion about commencing parenteral nutrition?	
Yes	5
No	0
Is there documented discussion about when to stop parenteral nutrition?	
Yes	0
No	5

GOJ = Gastro-oesophageal junction, ECOG = Eastern Cooperative Oncology Group.

### Data analysis

Analysis was carried out using a framework analysis approach [11] and coding managed using Microsoft Excel. All identifiable information, such as names and institutions, were first removed from transcripts. JM then became familiar with the data through immersive reading of all transcripts, identifying pertinent topics to include in the thematic framework. This process led to development of an initial framework (JM) which two authors (JM & SW) applied to three transcripts. Through discussion, the coding framework was revised and then applied to all transcripts by JM. The framework was iteratively revised throughout the process, with regular discussion between the two authors. The data was then charted for each theme and subtheme across all participants and summarised. Similar subthemes were combined to develop the final overarching themes and subthemes. Within each subtheme the data was grouped together where participants described a similar topic and varying opinions on this extrapolated. Exemplar quotations for each code assigned to a subtheme were extracted from the data to represent the views and experiences of participants (S2 Table).

This research is reported in line with the Standards for Reporting Qualitative Research [12] (S3 Checklist).

Participants were specifically asked what information they felt patients and carers required to make a decision to start or stop PN. Responses to this question were combined with participants’ reflections throughout the interviews to provide a set of recommendations to improve information provision and service delivery to facilitate decision-making around PN

### Ethical approval

Ethical approval was granted by North East- Newcastle & North Tyneside 2 Research Ethics Committee (20/NE/0277).

## Results

Twelve participants provided written consent to participate (6 patients, 6 carers) between January 2021 to June 2022. One patient died prior to interview so a total of 11 interviews (5 patients, 6 carers) were conducted. See Table 1 for patient characteristics. Of the 6 carer participants, 3 were currently caring for someone on HPN and 3 were previous carers.

Using framework analysis [11], five themes were identified:

- Factors affecting the decision: lack of choice and the importance of hope and advocacy
- Communication and information: whose role is it?
- Tackling discussions around benefits, risks and challenges of PN
- The reality of living with HPN
- Neglected conversations: stopping PN and advance care planning

### Factors affecting the decision: Lack of choice and the importance of hope and advocacy

This theme highlighted the limited options in providing nutritional support in advanced cancer where there was failure of the digestive tract and the inability to eat and drink sufficiently.

Most patients and carers felt there was no decision to be made around PN as there was no alternative form of nutrition available to them. The hope of survival led patients and carers to accept PN. One carer described the importance of PN to allow time for treatment to have an effect: *“it was made clear it was the only thing they could do to give her a chance”* (Carer 4), whilst a patient described feeling persuaded to have PN due to lack of alternatives: *“They [carer and Clinical Nurse Specialist] persuaded me, well why not try, because it’s the only option we have”* (Patient 3, HPN). Some patients noted the desire of their family for them to accept HPN. Another patient described declining PN as akin to suicide.

In some instances, the carer was influential in the patient receiving PN due to their own pre-existing knowledge of the intervention, leading some carers to request that PN was provided: *“Insisting and making sure parenteral nutrition was implemented, really came from me”* (Carer 1).

Patients and carers described the vulnerability of the patient at the time of decision-making and the feeling of safety offered by the hospital environment: *“I was quite vulnerable…when I was offered it was when I was at my lowest, you just take it don’t you*” (Patient 2, HPN).

Feelings of involvement in decision-making varied. Some patients did not feel involved in the decision-making process partly due to lack of options: “it just had to be” (Patient 4, HPN). Just one carer felt they were involved in decision-making.

### Communication and information giving: Whose role is it?

This theme identified the variety of ways in which people received information, how information was delivered and the inclusion of family and carers in the decision-making process.

Patients and carers were not always aware that PN could be considered if the patient was unable to manage adequate nutrition enterally. Some patients and carers noted the shock of being informed they may require PN as they had not been advised of this possibility earlier in the trajectory of their care: “*All this was like new to me and quite shocking, drastic really”* (Patient 2, HPN). Carers reflected on the lack of forward planning to prepare people for the potential need for PN, and that earlier discussions would have been preferable: *“It would have been better if they’d said look, you’re likely to run into these issues, we will see you at this point, this point, this point. We’ll assess where you’re going and then this might have to be the scenario. But that wasn’t the case. [He], I didn’t feel, was lined up to really appreciate that he was going to need parenteral nutrition”* (Carer 5).

There was variation in how much written information people wanted, with some reporting they received all the information they needed, whilst others felt more information would have been useful or interesting. For those who did receive written information, some commented that they could not remember the content, or that it was generic information. One carer noted although providing robust information may be best practice, it seemed unnecessary in the circumstances: “*It just felt that because it was an easy decision because it was so ‘life and death’, that was all a bit unnecessary really”* (Carer 2). Some received practical information before discharge on HPN around deliveries and what to expect at home, whereas others noted the involvement of the homecare company in providing information after discharge home.

Provision of information also varied according to the recruiting hospital. One patient commented that their team sent a video showing how to set up a multi-chamber bag, which they found to be useful. However, some participants reported looking for specific information online: “*I did an awful lot of research at the time on whether it would be a beneficial treatment to give an extra quality of life and I filled in all the gaps that no one had really talked to us about”* (Carer 2). Some carers also expressed the need to be cautious if joining online support groups and found them of limited benefit, one noting it may be useful for simple questions but not medical queries.

Carers noted that they were not actively involved in discussions or provided with written information, but rather received information via the patient. The COVID-19 pandemic impacted upon some carers not being present on the ward, though one noted being actively discouraged from being present at ward rounds even before visiting was restricted. Some noted the difficulties around communicating with the team during the pandemic *“the COVID situation made everything, including arranging or communicating the requirements for TPN in this case, so much harder”* (Carer 4). Only one carer reported the team contacted them directly by telephone to discuss the patient’s care during the pandemic.

Patients and carers typically talked positively about the support available once they were home with PN. People received support from homecare nurses through home visits or telephone contact: *“They’re always on the end of the phone, and they will always send a nurse to help me. And that’s good and reliable”* (Patient 4, HPN). People commented that they knew who to contact if they had questions. Most carers and some patients received training on administration of HPN once they were home and were keen to be trained to enable independence.

Participants noted that it was not always clear who took responsibility for the PN within the medical team, though several participants commented that the dietitian took primary responsibility. They also noted that the team were not always aware of who provided what information: *“we were told the same thing from numerous people, but there were certain things that no-one told us, because it was a no-one-quite-knows-who’s-telling-who-what type of situation”* (Carer 4).

Many professionals were acknowledged for their involvement in providing support and information around HPN including surgeons, oncologists, gastroenterologists, nurses and dietitians. Several participants noted their consultant recommended PN, whereas for others, PN discussions were initiated by their carer. Participants also noted the roles of the dietitian as providing information and taking responsibility for answering questions: *“He was very well supported. The dietitians there, they were able to talk him through how generally it would work”* (Carer 3). Some also had praise for the support provided by nursing staff. However, knowledge among team members was reported as variable. A patient with a multi-chamber bag at home noted *“The nurses here, still they don’t know what, what, how my TPN is set up, they still think I get it out the fridge”* (Patient 2, HPN), and another spoke of delegation within the team when they asked questions: *“the younger doctors or oncologist say oh you know I’ll leave it to the dietitian”* (Patient 1, inpatient PN).

### Tackling discussions around benefits, risks and challenges of PN

There was variation between participant’s experiences of whether benefits, risks and challenges of PN were discussed. Some reported that benefits were discussed, though others felt benefits were not discussed or they could not recall: *“the possible benefits of being on the feed…no, I don’t remember at all…I would remember…I’d be excited”* (Patient 2, HPN). Another patient commented that the benefit of survival was obvious. Similarly, some felt risks and challenges were discussed: *“We talked, a few times, about, at a certain point… it can exacerbate things like fluid retention”* (Carer 4). However, others could not recall explicit conversations around risk and some noted that managing or recognising potential complications was not discussed: *“I don’t think we really knew what to look for in terms of complications”* (Carer 5). One patient felt that the medical team might withhold information around risk: *“I don’t know they would ever tell you, what absolutely can go wrong at home”* (Patient 2, HPN).

Despite the variation reported in discussions around risks and challenges, patients and carers typically felt that the benefits of PN outweighed the potential negative impacts, particularly survival. One patient noted “not *starving to death is one benefit”* (Patient 5, HPN). A carer talked about PN in the wider context of dealing with the many aspects of living with advanced cancer: *“the complications related to TPN at that time would have been completely trivial in the whole scheme of things”* (Carer 2).

Several participants showed knowledge of some of the potential complications related to PN, mostly regarding infection risk. One patient noted organ dysfunction as a potential complication but could not remember details: *“The only one that I can remember came up was long-term potential damage to the kidney or liver – I can’t remember which – which could be a problem.”* (Patient 3, HPN).

### The reality of living with HPN

The logistics of arranging HPN were found to be challenging for many participants as this affected their hospital stay, potential transfers and the requirements for home.

Participants discussed several administrative aspects of preparing for and receiving HPN. The potential for transfer to a specialised intestinal failure centre to set-up HPN was noted. One patient established on HPN described feeling secure at the hospital and therefore reluctance to transfer hospital “*To go onto the parenteral nutrition, I’d have to go to [another hospital] for a period, yes, probably three, four weeks, something like that. I thought, I don’t want to do this*.” (Patient 3, HPN). Patients also discussed delayed discharge and inconsistent messages regarding this: *“at the beginning it was all about the TPN, getting it ready and it was taking 2 weeks and then 3 weeks, they were telling me, this only takes about 3 weeks…And then they tell me now, it will take about a month and then now, it will take up to 6 weeks or so”* (Patient 2, HPN).

Patients were typically made aware of the need for equipment to be delivered to their home prior to discharge, though were sometimes surprised by the space required “*we needed a whole roomful of space, because there were 40 boxes of stuff delivered”* (Carer 4).

Several patients and carers described the responsibilities of managing and ordering HPN supplies once home: *“So he has to keep a track on all his ancillaries, he has a spreadsheet”* (Carer 5). Patients also voiced frustration around being unable to access the equipment they required and unreliability of deliveries: *“it’s very important to chase deliveries because they more often don’t happen than do happen… we, on multiple occasions, are running out of equipment or those bags of food because they did not deliver what they should”* (Patient 5, HPN).

Patients and carers were typically keen to be trained to administer HPN if possible. The potential for training was often introduced by the homecare nurses once the patient was already at home. Noted benefits of being trained included independence, less nursing visits and flexibility around timing of giving PN: “*either me or my wife, they can train one of us to provide that so that we can have flexibility as to when to apply it rather than rely on the timing of the visit of the nurse, which made me very happy and we wanted to do that as quickly as possible”* (Patient 5, HPN). Carers would sometimes take responsibility for administering PN due to the practical issues encountered by patients: *“initially [he] had his line on his arm, so he couldn’t do it himself. He has now got a Hickman so he can do it all himself the majority of the time”* (Carer 2). Participants noted the training provided once home was rigorous and nursing support was available for those who required this.

Some participants noted the need to secure funding for HPN was communicated with them by healthcare professionals. In NHS England the care required for HPN is funded by the NHS, who contract commercial home care companies to provide these services [13]. The value of knowing funding requirements was questioned by one carer due to the anxiety this caused: “*the funding aspect of TPN. That was quite anxiety provoking, that thing of, what if the CCG* [Clinical Commissioning Group] *won’t fund it?”* (Carer 2).

Some carers reported a variation in practice around where HPN can be given, particularly in relation to hospices. One carer was advised PN could not be administered at their local hospice, whereas another reported HPN was delivered by the homecare nurse at a hospice in a different location. This impacted upon decision-making around preferred place of care: *“she was then left being afraid… thinking she wanted to go to a hospice because she didn’t think it would all go smoothly at home; but then being afraid to go to the hospice. Because the moment she got to the hospice, they’d try and stop feeding her”* (Carer 1).

### Neglected conversations: Stopping PN and advance care planning

The majority of patients and carers reported that stopping PN was not discussed, or they could not recall such discussions. No patients had a documented discussion around stopping PN in their medical records. Though most reported they had not had advance care planning discussions, one patient reported developing an advance care plan, whilst another instigated the discussion with their GP but was not sure what they wished to include. Of note, for those where advance care planning was considered, nutrition was not discussed: “*nothing about the TPN… I think that stays the same”* (Patient 2, HPN). One carer noted that the patient was not ready to have this discussion: *“advance care planning was something that we were aware of and they talked us through, but it was never something that [she] wanted to entertain for discussion”* (Carer 4).

The rationale for stopping or reducing PN was not clear for most, though some, with prior knowledge or experience, had some awareness of issues such as fluid overload or electrolyte abnormalities. One patient rationalised that people with some gastrointestinal function may make decisions around stopping based on factors such as quality of life, though felt this was less relevant when dependant on PN; *“If it’s a black and white you do or you don’t, it’s a bit less relevant, isn’t it? It’s quite obvious what would happen if you don’t have it”* (Patient 3, HPN). For one patient reliant on PN as their sole source of nutrition, the idea of stopping PN was introduced during the research interview which caused some distress; *“that made my heart skip. I thought, “Why would they stop it?” You think, “Oh my God, I’ve got nothing else*” (patient 3, HPN).

Carers reflected on their experiences of PN stopping, with three previous carers noting the decision was made to stop PN before the patient died. Some carers felt that it would be clear when the time was right to stop the PN as the patient approached the end of life. One current carer noted that PN may no longer provide a benefit if the patient was unconscious but did not feel this had been discussed.

### Recommendations

**Fig 1:**
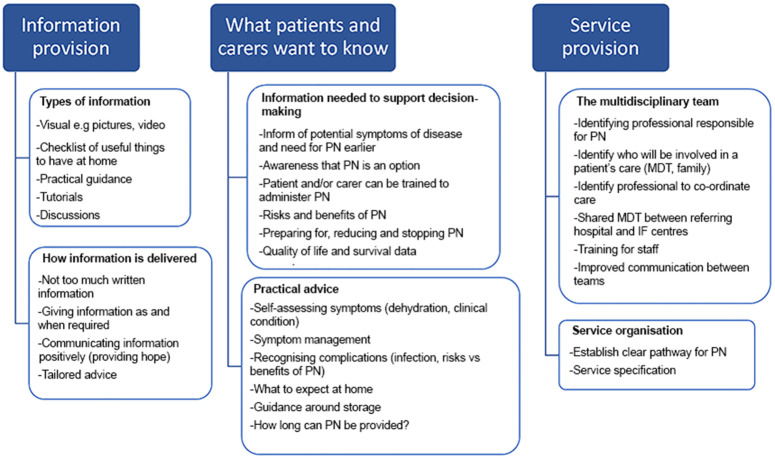
Patient and carer recommendations to improve decision-making around PN.

Participants suggestions on ways to improve decision-making around PN fell under three main categories; information provision, including mode of delivery, the information they required to make an informed decision and ways to improve service provision (Fig 1).

## Discussion

This is the first study to explore the information needs of people with advanced cancer, and their carers to facilitate decision-making around commencing or discontinuing PN. Findings indicate there are gaps in the provision of information around PN, including benefits, risks, challenges and discussion around stopping PN for both patients and carers. Participants identified areas for improved communication to support their decision-making and to improve service delivery.

The five themes identified in this study collectively illustrate how structural, emotional and informational factors shape the complexity of PN decision-making in advanced cancer. The perception among patients and carers that there was *no real choice* underscores how decisions were driven less by autonomous preference than by clinical necessity, at a time when patients were at their most vulnerable. Providing hope was identified as an important aspect of how information is delivered to patients and carers. It has been recognised that hope can also influence HCPs decision-making [14,15]. However, there can be a dichotomy for HCPs between providing hope and setting realistic expectations around the goals of PN. A recent study of people with malignant bowel obstruction (MBO) and recurrent gynaecological cancer found that patients value positive thinking, whereas HCPs are concerned with setting realistic expectations [16].

Variability in *communication and information giving* revealed systemic inconsistencies that disrupted shared decision-making, particularly where professional roles were unclear or carers were marginalised. The need for a clear service specification for PN was noted by participants, as well as the importance of identifying which members of the MDT are involved and which professional takes overall responsibility for overseeing PN. This was not always clear to patients or carers, and research has demonstrated that HCPs have differing views on who is responsible for decision-making around PN [8]. Communication between the managing team and the specialist intestinal failure service was also identified as an area for improvement. Having an allocated professional who oversees and co-ordinates care for people requiring HPN may improve service delivery. In a UK cancer centre, appointment of a nutrition nurse as a key point of contact was noted to improve communication and reduce time to discharge on HPN [8]. Cusimano et al. [17] suggest identifying a member of the MDT with experience and ability to liaise with departmental leaders and acknowledge the importance of building relationships with community service providers. Poor communication between HCPs has been identified in previous research as a potential barrier to facilitating timely discharge on HPN [8]. Lee et al. [18] have described the value of standardised assessment tools and documentation to allow transition between inpatient and outpatient services for those with MBO.

Guidelines [19] suggest that clear expectations of nutritional interventions in those with MBO should be discussed by a multidisciplinary team with patients and families. Despite guidance to involve carers in decision-making if the patient wishes [20], carers were not typically provided with information by HCPs or involved in decision-making. Similarly, a study among women with advanced ovarian cancer on HPN found that carers typically received information on an ad-hoc basis [21]. Carers described the responsibilities required to support the patient to receive HPN, including training to administer HPN, which many were keen to do. It is therefore essential that practical aspects of HPN are communicated to those who will help facilitate this. Patients making decisions around PN should be asked if they wish to involve a carer in discussions, and HCPs should ensure they receive information.

The importance of earlier conversations about the potential trajectory of disease and possible need for PN was also raised by patients and carers. It has been recognised that people with MBO referred for PN are often cachectic by time of referral and earlier nutritional care, integrated into patient pathways would be beneficial [22]. Early assessment and outpatient services to support people with gynaecological cancers and MBO have been developed in the UK and Canada, enabling earlier conversations around PN and goals of care [5,17].

One way to facilitate communication between HCPs, the patient and carers is through family meetings, allowing HCPs, the patient and family members/ carers to participate in shared decision-making and share information [23]. It is recognised that HCPs require advanced communication skills to facilitate a family meeting, particularly where sensitive discussions such as concerns around end-of-life and distress may arise [23]. Development and use of decision tools may also facilitate shared decision-making around PN.

Participants’ uncertainty about the *benefits, risks and challenges of PN* suggests that risk–benefit discussions are not only inconsistently delivered but may be strategically softened by clinicians attempting to balance realism with hope. Involvement of a specialist palliative care service may support these conversations. It has been suggested that early referral to palliative care services should be standard practice for those with advanced cancer and a new MBO diagnosis [17]. However, not all patients and carers wish to engage with palliative care services. A study evaluating supported self-management in MBO found that patients reported some resistance to accepting palliative care input due to perception that the service equated with death, however those who accepted input spoke positively on the service provided, including improved communication [17].

The theme concerning the *reality of living with HPN* highlights that the burdens of home PN—logistical, emotional and organisational—are often under-recognised during clinical decision-making, indicating a mismatch between professional assumptions and lived experience. A study by Sowerbutts et al. [21] reported that patients sometimes felt the difficulties of HPN were underplayed by HCPs. Similarly, one participant in our study felt HCPs may not discuss potential difficulties. Participants recommended that patients and carers should be made aware of what to expect at home and given practical advice around storage requirements. Many were only made aware they could be trained to administer PN once home, though felt that this would be useful to know at an earlier stage. Although patients and carers are encouraged to learn to connect and disconnect HPN, this should not delay discharge and most patients with advanced cancer and HPN will be expected to require nursing support [9]. Education for patients and carers to enable them to identify and manage symptoms, and recognise complications related to PN was recommended by participants. Lee et al. [18] note the importance of empowering patients and carers to manage symptoms and know when to seek support, recognising the high risk of recurrence of MBO among patients with gynaecological cancers specifically. Carers described the responsibilities required to support the patient to receive HPN, including training to administer HPN, which many were keen to do. It is therefore essential that practical aspects of HPN are communicated to those who will help facilitate this.

Finally, the absence of discussions around *stopping PN and advance care planning* reflects a broader discomfort with withdrawal of life-sustaining treatments. Multidisciplinary guidance around MBO suggests HPN should be stopped at the end of life (or not initiated) due to the risk of potential complications and prolonging suffering [19]. European guidance also states that PN is unlikely to provide benefit to most patients in the dying phase [24]. Despite this, the potential to reduce or the rationale for stopping PN is not adequately explained to patients or carers. Those who demonstrated an understanding that PN may be stopped had a previous knowledge of the intervention. This is echoed in a study by Sowerbutts et al. [21], where patients were surprised when the idea of stopping PN was raised by the researcher during interviews. Participants in this study indicated that options and reasons to reduce and stop PN should be discussed. We found that even those considering advanced directives had not had any conversations about nutrition as part of the discussion. As PN is a medical intervention [25], HCPs must ensure the benefits of PN outweigh the risks based on ethical principles of beneficence and non-maleficence. It is therefore important to explain which symptoms may indicate PN is causing harm, such as fluid overload. Furthermore, it is important to note that in the event of terminal hypometabolism, providing nutrition to meet the patients’ previous full nutritional requirements can induce metabolic distress [24], thus reduction of PN may be clinically indicated.

This study is not without limitations. We did not recruit any participants who declined PN, and therefore this study does not provide insight around the decision to decline PN.

The strengths of this work are that despite not achieving the stated aim to recruit 15 patients and 15 carers to the study, due to the recruitment timeframe and attrition, it did provide rich data with common themes generated through the interviews. It is acknowledged that collection of this data through interviews can be challenging for the participants as difficult topics may be discussed such as cessation of life sustaining support. The researchers are both experienced dietitians with specialist knowledge in improving decision-making around PN. On one occasion the researcher was known to the participant prior to the interview. We acknowledge that the professional background and rapport established during the interview may have shaped data collection, analysis and interpretation for this participant. To mitigate for this a third researcher (SW) who did not conduct interviews supported coding of the transcripts. This study was conducted in the UK and therefore results are not generalisable or transferable internationally to other care contexts.

Key points for future research of this type include recognition of the attrition that is anticipated in palliative care studies [26], consideration of ensuring all patient participants are asked if they have a carer to recruit to the study and including ethnicity data for patients and carers as culture can impact upon decision-making around PN [10].

## Conclusion

This study identifies the information that people with advanced cancer, and their carers need to make an informed decision to commence or discontinue PN. Starting and stopping HPN should be a shared decision-making process, but this study has demonstrated that this is not always the case and information for patients and carers is sometimes lacking. Patients and carers require clear communication around the benefits and potential risks associated with PN, as well as how to recognise and manage complications. Interventions, such as family meetings, identification of a key professional contact who co-ordinates PN and shared decision-making tools, are needed to improve communication and information provision for patients and carers. Improved communication between HCPs involved in HPN is also required and this could be facilitated through multidisciplinary meetings and standardised documents. Insights gained from this study may support the development of educational resources for HCPs.

## Supporting information

S1 Appendix Interview topic guides. (DOCX)

S2 Table Participant quotations. (DOCX)

S3 Checklist Standards for Reporting Qualitative Research. (DOCX)
