# Supplementary material for: Parenteral nutrition in advanced cancer: A qualitative study on decision-making and information needs of patients and carers
Source: PLoS One. 2026 Jun 2;21(6):e0350396. doi: 10.1371/journal.pone.0350396 (PMC13229323; doi:10.1371/journal.pone.0350396)
Supplement: S1 Appendix — (DOCX) [file pone.0350396.s001.docx]

**S1 Appendix: Interview topic guides**

**Interview topic guide- Patient**

Interview objective: To explore the information needs of patients with advanced cancer, when faced with the decision to commence and discontinue PN

**-Before starting confirm signed consent form received -**

- Confirm what the patient would like to be called throughout the interview (first name only)
- Start by providing a brief summary of the study, clarify what PN is, the purpose of the interview, expected duration
- Advise patient that they can take breaks at any time, and stop the interview if they wish
- Advise patient that the researcher cannot provide any clinical advice
- Advise that the interview will be recorded and transcribed

**-Start recording-**

- Can you advise if you are currently on parenteral nutrition, or if you are deciding whether to start parenteral nutrition

*If receiving PN use question set 1*

*If deciding whether to start PN use question set 2*

**Question set 1 – for patients receiving PN**

1. **Can you tell me what you know about parenteral nutrition?**
2. **Can you tell me about your experience of deciding to start parenteral nutrition?**
   1. What were the reasons you started parenteral nutrition?
   2. Were specific goals of parenteral nutrition discussed?
   3. When did you start parenteral nutrition?
   4. Who did you speak to about deciding to start parenteral nutrition? e.g. which healthcare professionals/ family/ friends
   5. How did you feel during the decision-making process?
   6. What made you decide to start parenteral nutrition?
3. **What information were you given in order to make the decision to have parenteral nutrition?**
   1. Who discussed this with you first?
   2. What were you told? Were the consequences of accepting or declining PN discussed?
   3. **Home PN only:** were you told about what to expect e.g. hospital visits, equipment, nursing input at home
4. **Did anyone talk to you about the potential benefits of parenteral nutrition?**
5. **Did anyone talk to you about the potential risks of parenteral nutrition?**
   1. Did anyone talk about why parenteral nutrition might be stopped?
   2. Did you receive any information on how any complications might be managed?
   3. Did anyone discuss an Advance Care Plan with you? This is a plan you make with your doctors around your wishes for your care in the future.
6. **Do you feel you were given all the information you needed to make the decision to have parenteral nutrition?**
   1. Were you given verbal and written information? Was this useful?
   2. Would you have preferred the information to be given differently? If so, how would you like to be given information?
   3. How much information did you want?
   4. Did you look for information anywhere else e.g. internet
   5. With hindsight, was there anything you feel you should have been told about that you were not? Would this information have changed your decision?
   6. Did the information you received prepare you fully?
   7. Did you understand the information you were given?
   8. Were you given the chance to ask questions?
7. **Did you feel that you were involved in the decision to have parenteral nutrition?**
8. **What information do you think people need when they are deciding whether to start or stop parenteral nutrition?**

**Finally, is there something we have not discussed that you would like to add?**

**Thank you for taking part today.**

**Next steps:**

Once all of the interviews have been carried out the research will be written up so it can be published in a journal. We will put a summary of the study of the Royal Marsden website which you will be able to access.

**Sign posting**- If any issues raised send hand-out on support groups by email or post

**Question set 2- for patients considering PN**

1. **Can you tell me what you know about parenteral nutrition?**
2. **Can you tell me about your experience so far in deciding whether or not to have parenteral nutrition?**
   1. What were the circumstances that led to you making this decision?
   2. Have specific goals of parenteral nutrition discussed?
   3. Who have you spoken to? e.g. which healthcare professionals/ family/ friends
   4. How do you feel about making this decision?
3. **What information have you been given to help you make the decision on whether or not to have parenteral nutrition?**
   1. Who discussed this with you first?
   2. What have you been told? Have the consequences of accepting or declining PN been discussed?
   3. Have you been given written and verbal information?
4. **Has anyone explained the potential benefits of parenteral nutrition to you?**
5. **Has anyone explained the potential risks of parenteral nutrition to you?**
   1. Has anyone spoken with you about why parenteral nutrition might be stopped?
   2. Have you received any information on how any complications might be managed?
   3. Do you have an Advance Care Plan, or has this been discussed with you? This is a plan you make with your doctors around your wishes for your care in the future.
6. **Do you feel you have all the information you need in order to make a decision around parenteral nutrition?**
   1. Was the information you have received e.g. verbal, written useful?
   2. Would you have preferred the information to be given differently? If so, how would you like to be given information?
   3. How much information would you like to receive?
   4. Have you looked for information anywhere else? e.g. internet
   5. Have you understood the information you have been given?
   6. Have you been given the opportunity to ask questions?
7. **Do you feel you have been involved in the decision making process around parenteral nutrition?**
8. **What information do you think people need when making a decision on whether or not to start or stop parenteral nutrition?**

**Finally, is there something we have not discussed that you would like to add?**

**Thank you for taking part today.**

**Next steps:**

Once all of the interviews have been carried out the research will be written up so it can be published in a journal. We will put a summary of the study of the Royal Marsden website which you will be able to access.

**Sign posting**- If any issues raised send hand-out on support groups by email or post

**Interview topic guide- Carers**

Interview objective: To explore the information needs of people caring for patients with advanced cancer, when supporting the person they care for to make a decision to commence and discontinue PN

-**Before starting - confirm signed consent form received-**

- Confirm what the participant would like to be called throughout the interview (first name only)
- Start by providing a brief summary of the study, clarify what PN is, and the purpose of the interview
- Advise participant that they can take breaks at any time, and stop the interview if they wish
- Advise participant that the researcher cannot provide any clinical advice
- Advise that the interview will be recorded

**-Start recording-**

- Can you advise if you are current a current carer or have previously cared for someone with advanced cancer on parenteral nutrition?
- **Current carers only:** Can you advise if the person you care for is currently on parenteral nutrition, or deciding whether to start parenteral nutrition?

*If carer previously cared for someone receiving PN or currently cares for someone receiving PN use question set 1*

*If caring for a person deciding whether to start PN use question set 2*

- Can you advise what name you would like us to refer to the person you care/d for throughout the interview? Please provide a first name only. If you prefer us not to use their name, we will refer to them as ‘the person you care/d for’

**Question set 1- care/d for patient receiving PN**

1. **Can you tell me about your experience of supporting [insert name] to make a decision around parenteral nutrition?**
   1. What were the circumstances that led to making this decision?
   2. Who did you speak to? e.g. healthcare professionals/ family/friends
   3. How did you feel during the decision-making process?
   4. What made the person you care/d for decide to start parenteral nutrition?
   5. Did you and [insert name] have different opinions about parenteral nutrition?
2. **What information were you given to support [insert name] to make the decision to have parenteral nutrition?**
   1. Who discussed this with you first, and when?
   2. What were you told?
3. **Did anyone explain the benefits of parenteral nutrition to you and [insert name]?**
4. **Did anyone explain the risks of parenteral nutrition to you and [insert name]?**
   1. Did anyone talk about why parenteral nutrition might be stopped?
   2. Did you and [insert name] receive any information on how any complications might be managed?
   3. Did anyone discuss an Advance Care Plan with you and [insert name]? This is a plan you make with your doctors around a patient’s wishes for their care in the future.
5. **Do you feel you were given all the information you needed to support [insert name] to make a decision to have parenteral nutrition?**
   1. Were you given verbal and written information? Was this useful?
   2. Would you have preferred the information to be given differently? If so, how would you like to be given information?
   3. How much information did you want?
   4. Did you look for information anywhere else? e.g. internet
   5. With hindsight, was there anything you feel you, and [insert name], should have been told about that you were not?
   6. Did the information you received prepare you fully?
   7. Did you understand the information you were given?
   8. Were you given the chance to ask questions?
6. **Did you feel that you were included in the decision making around [insert name] starting parenteral nutrition?**
7. **What information do you think people need when they are deciding whether or not to start or stop parenteral nutrition?**

**Finally, is there something we have not discussed today that you would like to add?**

**Thank you for taking part.**

**Next steps:**

Once all of the interviews have been carried out the research will be written up so it can be published in a journal. We will put a summary of the study of the Royal Marsden website which you will be able to access.

**Sign posting**- If any issues raised send hand-out on support groups by email or post

**Question set 2- carer for patient considering PN**

1. **Can you tell me about your experience so far in supporting [insert name] to make a decision about whether or not to have parenteral nutrition?**
   1. What were the circumstances that led to making this decision?
   2. Who have you spoken to? e.g. healthcare professionals/ family/ friends
   3. How do you feel about supporting {insert name] to make this decision?
2. **What information have you and [insert name] been given to help you make the decision on whether or not to have parenteral nutrition?**
   1. Who discussed this with you and [insert name] first?
   2. What have you and [insert name] been told?
   3. Have you and [insert name] been given written and verbal information?
3. **Has anyone explained the benefits of parenteral nutrition to you and [insert name]?**
4. **Has anyone explained the risks of parenteral nutrition to you and [insert name]?**
   1. Has anyone spoken with you and [insert name] about why parenteral nutrition might be stopped?
   2. Have you and [insert name] received any information on how any complications might be managed?
   3. Does [insert name] have an Advance Care Plan, or has this been discussed with you and [insert name]? This is a plan a patient makes with their doctors around their wishes for their care in the future.
5. **Do you feel you have all the information you need in order to support [insert name] to make a decision around parenteral nutrition?**
   1. Was the information you and [insert name] have received e.g. verbal, written useful?
   2. Would you have preferred the information to be given differently? If so, how would you like to be given information?
   3. How much information would you like to receive?
   4. Have you looked for information anywhere else? e.g. internet
   5. Have you understood the information you and [insert name] have been given?
   6. Have you been given the opportunity to ask questions?
6. **Do you feel you have been involved in the decision making process around parenteral nutrition?**
7. **What information do you think people need when making a decision on whether or not to start or stop parenteral nutrition?**

**Finally, is there something we have not discussed that you would like to add?**

**Thank you for taking part today.**

**Next steps:**

Once all of the interviews have been carried out the research will be written up so it can be published in a journal. We will put a summary of the study of the Royal Marsden website which you will be able to access.

**Sign posting**- If any issues raised send hand-out on support groups by email or post
