# Supplementary material for: Parenteral nutrition in advanced cancer: A qualitative study on decision-making and information needs of patients and carers
Source: PLoS One. 2026 Jun 2;21(6):e0350396. doi: 10.1371/journal.pone.0350396 (PMC13229323; doi:10.1371/journal.pone.0350396)
Supplement: S2 Table — (DOCX) [file pone.0350396.s002.docx]

**S2 Table: Participant quotations**

**Factors affecting the decision: no real choice**

| **Subtheme and description** | **Code** | **Example quotations** |
| --- | --- | --- |
| **There was no other option**  Patients and carers described lack of choice since PN was the only way to provide adequate nutrition. Participants described PN in terms of survival and providing a chance for treatment to work. | There was no decision to be made  No choice was given  Inability to eat or drink  Desire for nutrition  Persuaded to accept PN  Nutrition required to receive treatment | *“it wasn’t really a choice. There was only, really, one course” (Carer 4)*  *“they don’t really give you a choice. This is everything… on their terms” (patient 2, HPN)*  *“although he could technically eat, nothing would have stayed down” (Carer 2)*  *“she wanted to be fed” (Carer 1)*  *“They [carer and CNS] persuaded me, well why not try, because it’s the only option we have” (patient 3, HPN)*  *“this was the only thing that was going to give a chance of responding to treatment, so you’ve got to do it” (Carer 4)* |
| **Influence of the carer**  The carer voice was important in some cases for ensuring the patient received PN. Several carers had pre-existing knowledge of PN. | Carer had pre-existing knowledge of PN  Carer requested PN  Carer keen for the patient to receive PN | *“I remember thinking to myself, he’ll need home PN, no doubt about that” (Carer 5)*  *“Insisting and making sure parenteral nutrition was implemented, really came from me” (Carer 1)*  *“my daughter, she was, well she was quite adamant” (Patient 2, HPN)* |
| **Uncertainty and vulnerability**  Participants described the vulnerability of the patient at the time of making decisions around PN and trust in their healthcare team. This introduced further vulnerability when transfer to another hospital or local A&E attendance was necessary | Patient vulnerability  Patient did not want to move to another hospital  Patient uncertain about PN | *“I was quite vulnerable…when I was offered it was when I was at my lowest, you just take it don’t you” (Patient 2, HPN)*  *“when you’re in hospital you get to a point, you feel safe and secure where you are. I was there, I knew the people, I knew the routines, I’m close to my family” (Patient 3, HPN)*  *“I wasn’t one hundred percent sure” (Patient 2, HPN)* |
| **Involvement in decision-making**  There was variation in how involved both patients and carers felt in the decision-making process. | Patient not part of decision-making  Patient made decision with support  Carer didn’t feel part of decision-making | “do you feel you were involved…in the decision-making process around going home with your parenteral nutrition?”  *“not really” (Patient 4, HPN)*  *“I was given the facts and I was given the emotional support as well, but there was no doubt I made the decision” (Patient 3, HPN)*  *“I definitely didn’t feel part of the decision-making” (Carer 4)* |
| **Differences in opinion**  Patients, carers and healthcare professionals sometimes had different opinions about the best options for nutrition support. This could be a source of friction. | Difference in opinion from healthcare team  Patient reluctant to have PN  Friction between patient and carer | *“I never felt I needed it, so I sort of went for an NJ tube insertion” (Patient 1, inpatient PN)*  *“I’ll be honest, I really wasn’t very keen. I’d really had enough at that point” (Patient 3, HPN)*  *“I didn’t even tell him about it [home PN] because at that time it was causing a lot of friction between us” (Carer 5)* |

**Communication and information: whose role is it?**

| **Subtheme and description** | **Code** | **Example quotations** |
| --- | --- | --- |
| **The unknown**  Participants were not informed that PN may be required early in the disease trajectory and therefore the suggestion to start PN came as a shock at the time it was the only option left. | PN came as a surprise  Lack of forward planning  Unaware of options | “*All this was like new to me and quite shocking, drastic really” (Patient 2, HPN)*  *“It would have been better if they’d said look, you’re likely to run into these issues, we will see you at this point, this point, this point. We’ll assess where you’re going and then this might have to be the scenario. But that wasn’t the case. [He], I didn’t feel, was lined up to really appreciate that he was going to need parenteral nutrition” (Carer 5)*  *“I don’t think I would have even known about parenteral nutrition. If I hadn’t have known it existed I don’t think I would have even known that it was an option” (Carer 5)* |
| **Written information**  Receiving of written information was variable. Some felt it had limited usefulness whilst others could not remember the content. Some felt more written information would have been useful. Many on HPN noted receiving written information from the HPN company. | Did not receive written information  Did receive written information  Could not remember content  Limited usefulness  Would have found useful | *“I don’t think I got any written information per se” (Patient 1, inpatient PN)*  *“We were given general handouts, if you like. You know the whole leaflet-type thing” (Carer 4)*  *“I don’t even remember what I read, was, I don’t think I finished reading it yet” (Patient 2, HPN)*  *“The written information that you got, has it been useful to you?” “Well only the phone numbers, really” (Patient 4, HPN)*  *“it would be nice to have more written information” (Patient 1, inpatient PN)* |
| **Level of information provided**  Some participants felt well informed. Some felt too much information was provided in terms of trying to process the information, and that information provided would not have changed the decision to have PN as there was no alternative. Whereas others would have appreciated more information. | Too much information  Felt information not necessary  Felt well informed  Not all information was provided  Made their own practical decisions  Forgot information provided  Received practical information | *“It was too much for me to take on …to read all these then” (Patient 2, HPN)*  *“so I think if you look at it purely in terms of best practice, you would like to think, wouldn't you, that there's a meaningful conversation where someone takes the time to really go into all the in-depth nitty-gritty of "Well, this can happen, that can happen. Oh, look at this video. Look at this" and have amazing resources available for the patient. It just felt that because it was an easy decision because it was so 'life and death', that was all a bit unnecessary, really” (Carer 2)*  *“Before, I think I did have enough information” (Patient 5, HPN)*  *“It would have been nice to have- I'm very much one of those people who needs to know how things work and why they work. I think if I’d had all of that information, but with having said that, yes, it would have been nice to have had it. Yes, having that knowledge would have been nice. Would it have benefited? Would it have made any difference to what we had done? Absolutely not It would just be one of those things that it would be nice to know” (Carer 3)*  *“Running it overnight, for example, so that didn’t interfere with the day and things, that was things that we had decided ourselves” (Carer 3)*  *“the questions that you’ve asked, I’ve forgotten” (Patient 1, inpatient PN)*  *“At [the hospital], they explained – really to my wife, but also me in more detail… "This is what it's going to involve, deliveries will be this often," and just the practicalities of it I suppose” (Patient 3, HPN)* |
| **Communication with carers**  Carers were not specifically provided with information. Information was relayed by the patient or requested from the healthcare team by the carer. | Carer not involved in discussions  Carer did not receive information | *“I don’t remember any discussions with me present” (Carer 5)*  *“So I didn’t get a great deal of information, other than what [she] gave me” (Carer 4)* |
| **Support available**  Participants spoke positively of the nursing care provided on HPN and availability of telephone and email support. They typically felt they knew who to contact if they had concerns. Some were trained on how to administer the PN once home. | Training provided  Nurses would visit if needed  Praise for PN company  Telephone and email support  Given a chance to ask questions  Knew where to get support | *“They trained, well, me and wife in the connecting and disconnecting and they trained my wife in the dressing changes” (Patient 3, HPN)*  *“They're always on the end of the phone, and they will always send a nurse to help me. And that's good and reliable” (Patient 4, HPN)*  *“you always knew who you could phone day or night with it, really. So it felt like you were very well supported from that point of view” (Carer 2)*  *“if I’m ever worried about anything I just contact them and it’s not a problem” (Carer 6)*  *“I had access to people as well if I had any questions” (Carer 6)*  *“If I’ve got a question I can ring someone, I know who to ring for that particular question” (Patient 4, HPN)* |
| **The multidisciplinary team**  Participants were not always clear which professional took responsibility for the PN. Dietitians, consultants, surgeons and nurses were noted as professionals with involvement. Care from the team was not always co-ordinated as it was not clear who had discussed what aspects of care. HCPs did not always have a thorough knowledge of PN such as indications in advanced cancer or how PN is delivered. | Not clear who provides what information  Not clear who was responsible for nutrition  Dietitian involvement  Consultant involvement  Praise for nurses  Surgical involvement  Lack of HCP knowledge of PN  Information given sporadically | *“we were told the same thing from numerous people, but there were certain things that no-one told us, because it was a no-one-quite-knows-who’s-telling-who-what type of situation” (Carer 4)*  *“So, there were three different people who might have been responsible for her nutrition, but actually none of them got a grip on the situation” (Carer 1)*  *“He was very well supported. The dietitians there, they were able to talk him through how generally it would work” (Carer 3)*  *“He used to give us lots and lots of information, but that was predominantly aimed at [his] treatment” (Carer 3)*  *“people were ready to discuss, and some of the nurses were very good” (Patient 4, HPN)*  *“her surgeon, he did call me, a few times, about it, so there was a discussion with him and one of his team members” (Carer 4)*  *“The nurses here, still they don’t know what, what, how my TPN is set up, they still think I get it out the fridge” (Patient 2, HPN)*  *“so basically I got information here and there from the doctors, emm, but later”(Patient 2, HPN)* |
| **Sources of information**  Some looked for further information online. Participants noted the importance of exercising caution with online support groups, finding these to be of limited benefit. | Looked for information online  Did not look for information online  Support groups  Video | *“It was up to me to, I suppose, do Googling to look at "What use is TPN in cancer?" and draw my own conclusions from that” (Carer 2)*  *“my daughter did... I think my daughter did …… I didn’t” (Patient 2, HPN)*  *“one thinks these things are helpful in reality, but actually, in this instance, they weren't because everyone was being so alarmist” (Carer 2)*  *“the team at [the hospital] sent me on a video as to how to make the multichamber bag work” (Carer 6)* |

**Tackling discussions around benefits, risks and challenges of PN**

| **Subtheme and description** | **Code** | **Example quotations** |
| --- | --- | --- |
| **Discussion around benefits**  Benefits of PN were discussed with some participants though not all. Some could not remember if benefits were discussed. | Benefits not discussed  Unable to remember if discussed  Benefits discussed | *“no, what benefits are there?” (Patient 4, HPN)*  *“the possible benefits of being on the feed…no, I don’t remember at all…I would remember…I’d be excited” (Patient 2, HPN)*  *“yes, the benefits were really weighed in” (Carer 4)* |
| **Discussion around risks and challenges**  Discussions around risks and challenges varied, some felt this was addressed whilst others did not. Some participants noted not knowing what to look for in terms of complications of PN or how to manage them. | Managing complications not discussed  Risks not discussed  Felt medical team would not discuss risks  Risks discussed | *“I don’t think we really knew what to look for in terms of complications” (Carer 5)*  *“I can’t recall an explicit conversation, sitting down and going through it in detail” (Carer 2)*  *“I don’t know they would ever tell you, what absolutely can go wrong at home” (Patient 2, HPN)*  *“We talked, a few times, about, at a certain point…It can exacerbate things like fluid retention. It can exacerbate oedema in patients” (Carer 4)* |
| **Benefits perceived to outweigh the risks**  Most participants felt any complications of PN would be outweighed by the benefits, namely survival. | Survival  Complications trivial | “*like I said, the survival and not starving to death is one benefit” (Patient 5, HPN)*  *“the complications related to TPN at that time would have been completely trivial in the whole scheme of things, really” (Carer 2)* |
| **Awareness of potential complications**  Some participants were aware of some of the potential complications of PN, though did not have an overarching knowledge of these. | Infection risk  Organ dysfunction | *“infection because of the line. That was one possible side effect that they explained could happen.” (Patient 5, HPN)*  *“The only one that I can remember came up was long-term potential damage to the kidney or liver – I can't remember which – which could be a problem.” (Patient 3, HPN)* |

**The reality of living with HPN**

| **Subtheme and description** | **Code** | **Example quotations** |
| --- | --- | --- |
| **Aims of HPN**  Participants noted different aims of HPN including to allow them to get home, survival and to support treatment. | To enable patient to go home  Survival  To support treatment | *“you don’t want to be in any longer than you have to. Obviously, with [her] I think they, probably, also, felt, deep down, that these were precious moments, and to be alone, in hospital, for them probably wasn’t the best for anybody”* (Carer 4)  *“I knew that the aim was for me to survive” (Patient 2, HPN)*  *“we were sent home, with TPN, to try to give immunotherapy a chance to carry on working” (Carer 4)* |
| **Administration involved with HPN**  Participants were aware they would require equipment at home and a specialist team to set this up. They noted the volume of administration involved once on HPN such as monitoring their deliveries to ensure they had all they needed, this was a cause of anxiety. Participants noted the unreliability of the delivery system and having to chase deliveries. | Involvement of intestinal failure centre  Delayed discharge  Informed of equipment required  Patient in charge of inventory  Availability of HPN  Unreliable system | *“I might have to wait until there’s another centre able to supply what I’m getting in the hospital” (Patient 1, inpatient PN)*  *“at the beginning it was all about the TPN, getting it ready and it was taking 2 weeks and then 3 weeks, they were telling me, this only takes about 3 weeks…And then they tell me now, it will take about a month and then now, it will take up to 6 weeks or so, it seems like every week they are telling me different things…So there was never a set a date when I will actually get the TPN…and then it was like 2 months…I had to wait 2 months”* *(Patient 2, HPN)*  *“did people speak to you about things like the nursing, um, input at home or the type of equipment and things that could be…” “oh yeah, sorry, yes they did yeah” (Patient 1, inpatient PN)*  *“So he has to keep a track on all his ancillaries, he has a spreadsheet. He's a very organised person but he has a spreadsheet for all his ancillaries they bring him each month. He needs to say exactly what he needs and there’s loads of stuff” (Carer 5)*  *“please make the bags available easily, (Laughter) because it was a great worry, my daughter been in the hospital to get them for me, and back and forward into [hospital]” (Patient 4, HPN)*  *“it’s very important to chase deliveries because they more often don’t happen than do happen. For some reason the delivery company is very unreliable and we, on multiple occasions, are running out of equipment or those bags of food because they did not deliver what they should deliver even though we agreed on the phone with the nurses that was what should be delivered” (Patient 5, HPN)* |
| **Storage**  Participants were aware of the need for equipment and space for storage. However, were surprised at the amount of space needed for all their supplies. | Need space for the equipment and ancillaries  Equipment well maintained | *“Off-the-shelf TPN can be stored in boxes, and [hers] was off the shelf. But then, of course,* *we needed a whole roomful of space, because there were 40 boxes of stuff delivered” (Carer 4)*  *“The medical fridge that they provide is very well looked after. Mine was serviced, automatically serviced after so long. And then it did go wrong, something went wrong with it one day, I discovered that it wasn't as cold as it should be. The next day they delivered a new fridge. I mean, they were really very quick off the mark. And it was all done efficiently and quickly” (Patient 4, HPN)* |
| **Funding**  Participants noted awaiting confirmation of funding through the CCG for HPN, and the resulting anxiety. The the value of knowing funding needed to be secured was questioned. | Funding has to be confirmed  Didn’t take long  Concerns about funding | *“I think the only thing of concern was this thing about "Oh, would the CCG fund it?" because it meant that the oncologists had to declare that he would survive a certain amount of time” (Carer 2)*  *“obviously they had to sort the funding. That actually, in the great scheme of things, didn't take that long” (Patient 3, HPN)*  *“I mean, in the end, the funding was never an issue, really. It all happened in the background without us knowing of any of the ins and outs of that… I suppose the other thing, and maybe I haven't said, is* *the funding aspect of TPN. That was quite anxiety provoking, that thing of: "What if the CCG won't fund it?" (Carer 2)* |
| **Training around PN**  Participants were typically keen to be trained to administer PN (patient and/or carer) to have some autonomy and control over their schedule. The role of the homecare nurses was central as they provided training and support. The training provided once home was thorough. | Patient and/ or carer had training to administer PN  Training was rigorous  Carer supported with HPN administration  Homecare nurses advised regarding training  Keen to self-administer  Unaware of flexibility with HPN  Nursing support available | *“With time,* *either me or my wife, they can train one of us to provide that so that we can have flexibility as to when to apply it rather than rely on the timing of the visit of the nurse, which made me very happy and we wanted to do that as quickly as possible” (Patient 5, HPN)*  *“I then underwent their training programme, which was fairly rigorous. I had to be seen to connect him and disconnect him a certain number of times and get signed off for doing it and go through all of their protocols” (Carer 2)*  *“For a few years I was doing the plugging in, because initially [he] had his line on his arm, so he couldn’t do it himself”* *(Carer 2)*  *“they decided quite soon that I was capable of doing it myself, which was a great improvement because I could choose my own time” (Patient 4, HPN)*  *“he always wanted to do the PN himself providing he was well” (Carer 5)*  *“I think a lot of people probably think it’s quite set in stone. They wouldn’t know that maybe the formula can be changed. Or, if he starts stacking on loads of weight and it’s making you feel uncomfortable then we’ll look at reducing down the number of times a week you’re having it” (Carer 5)*  *“they explained that there’s going to be a nurse coming in every day, in the morning and the evening” (Patient 5, HPN)* |
| **Preferred place of care**  There is variation in practice around where PN can be provided, particularly in relation to hospices which is down to each individual hospice | Advised unable to have HPN at hospice | *“when I spoke to the palliative care consultant at the hospice – this is not [the hospital] palliative care, this is for [location]– she said they would not give parenteral nutrition in the hospice” (Carer 1)* |

**Neglected conversations: Stopping PN and advance care planning**

| **Subtheme and description** | **Code** | **Example quotations** |
| --- | --- | --- |
| **Lack of discussion about stopping PN**  Most participants felt reasons for stopping PN were not discussed. The idea of stopping caused alarm for some participants. Others with some knowledge of PN felt it would be clear when to stop PN towards the end of life. | Stopping PN not discussed  Reasons for stopping PN not clear  Concerns over stopping PN  Implicit understanding that PN would stop eventually | “*no, there has not been any conversation about whether it would ever need to be withdrawn or anything like that, or what implications, what sort of thing would make that decision for us” (Carer 3)*  “I don’t really understand when do you say we’re stopping the PN? Is there a consensus so they just keep the PN running or is it just that they keep it as long as it’s not causing any issues?” (Carer 5)  *“that made my heart skip. I thought, "Why would they stop it?" You think, "Oh my God, I've got nothing else." (Patient 3, HPN)*  “*I can't remember, but I think there was a tacit understanding that at some point in time then, yes, we would stop it, and certainly I knew that that would be the case”* *(Carer 2)* |
| **The decision to stop PN**  The decision to stop PN arose for different reasons, such as pausing PN due to electrolyte abnormalities or the patients decision to stop PN towards the end of life. Carers described this as a clear decision to make when the patient neared the end of life. | PN paused by medical team  Patient made the decision to stop PN  Clarity on when to stop PN  Decision should come from the patient | *“my electrolytes…sometimes they were a bit high... yeah, so they stopped it” (Patient 1, inpatient PN)*  *“there was a moment, when she realised and I realised, that actually, this is it, she’s not getting out of hospital again. And she made that decision” (Carer 1)*  *“before it became obvious, it was unclear to me how you'd make that decision. I sort of envisaged in my head that he'd probably be on it right up to the moment he died, but actually, when the time was right to stop it, it was clear that "we're on the road to nowhere with this. He is dying” (Carer 2)*  *“I think this decision really has to come from the patient” (Carer 1)* |
| **Advance care planning**  Experiences with Advance Care Planning varied. Some were aware of ACP and working on this, whilst others were not aware of ACP or did not wish to discuss it. Nutrition was not a part of ACP discussions. | ACP discussions in progress  ACP not discussed  Advanced directives on nutrition not discussed  Undecided regarding ACP  Patient not willing to engage in discussion | *“we’re working on it” (Patient 2, HPN)*  *Has that ever been discussed with you?*  *“no” (Patient 5, HPN)*  *“but* *nothing about the TPN… I think that stays the same” (Patient 2, HPN)*  *“I haven’t put one in place because I haven’t really got my mind around quite what I want from it. Yes, I have discussed it, but with my GP at my instigation” (Patient 3, HPN)*  *“the advance care planning was something that we were aware of and they talked us through, but it was never something that [she] wanted to entertain for discussion” (Carer 4)* |
